# Can pharmacological receptor tyrosine kinase inhibitors sensitize poor outcome breast tumors to immune-based therapies?

**DOI:** 10.3389/fonc.2013.00023

**Published:** 2013-02-13

**Authors:** Josie Ursini-Siegel

**Affiliations:** Lady Davis Institute for Medical ResearchMontreal, QC, Canada

**Keywords:** breast cancer, cytotoxic T lymphocyte, immune suppression, HER2/basal, receptor tyrosine kinase

## Abstract

Receptor tyrosine kinases (RTKs) drive breast cancer progression, particularly in human epidermal growth factor receptor 2 and basal tumors, the two worst prognosis subtypes. Tumor cells recruit host stromal components, including immune cells, which strongly influence disease progression. This has been studied in human breast cancer and translated to murine models of breast cancer. Stromal immune components including cytotoxic T lymphocytes (CTLs) and natural killer cells, destroy cancer cells through a process termed immune surveillance. Unfortunately, clinically detectable tumors escape these immune protective effects through their ability to limit the infiltration, activation, and/or survival of CTLs in breast tumors. The immunosuppressed state of established tumors limits the success rate of immune-based therapies, and possibly other therapeutic modalities that depend on host immunity. Published studies demonstrate that RTKs facilitate breast cancer progression, in part, by establishing immune suppression. This raises the intriguing possibility that pharmacological RTK inhibitors may be exploited to sensitize breast cancer patients to immune-based therapies.

## BREAST CANCER HETEROGENEITY

Major progress has been made since the recognition that breast cancer is a heterogeneous disease, which is molecularly defined by five subtypes based on unique transcriptional profiles and expression of the estrogen and progesterone receptors (ER and PR) and the human epidermal growth factor receptor 2 (HER2) receptor tyrosine kinase (RTK). This includes normal, luminal A/B, HER2, and basal/triple negative (ER^-^/PR^-^/HER2^-^) cancers (Perou et al., 2000; van de Vijver et al., 2002; van ’t Veer et al., 2002). Within these subtypes, HER2 and basal breast cancers represent 30–40% of all newly diagnosed cases and are associated with the worst outcome. The basal subtype is stratified into two groups: (1) basal A tumors, which co-express basal (CK5/6, CK14) and luminal (CK8/18) cytokeratins and (2) basal B or “claudin-low” tumors, which lost their luminal characteristics (Neve et al., 2006), including reduced expression of tight junctional proteins called claudins. These tumors are enriched in genes associated with a stem cell phenotype and an epithelial to mesenchymal transition (Prat et al., 2010; Taube et al., 2010).

Amplification, overexpression or activation of RTK promotes disease progression in HER2 and basal breast cancers. HER2 [also known as v-erb-b2 erythroblastic leukemia viral oncogene homolog 2 (ErbB2)] amplification is a defining feature of the HER2 subtype, correlating with poor outcome (Slamon et al., 1989). Overexpression of ErbB2 in the mammary glands of transgenic mice leads to tumor development (Muller et al., 1988). The Met RTK is a clinically relevant biomarker of poor outcome basal breast cancers and drives the formation of mouse mammary tumors with distinct histopathologies, a subset of which resemble the basal subtype (Ponzo et al., 2009). Personalized therapy targeting HER2 (trastuzumab), coupled with chemotherapy, increases disease-free survival of early stage HER2^+^ breast cancer patients. Nonetheless, in the metastatic setting, these patients are refractory to such combination therapies. While Met-targeted therapies (Tivantinib) are in clinical trials in triple negative breast cancer patients, chemotherapy remains the standard of care for this subtype. This highlights the need to better understand factors that influence responsiveness to targeted therapies. Herein, we discuss whether RTK therapies can locally re-activate anti-tumor immunity and sensitize poor outcome breast cancers to immunotherapy.

## PARADOXICAL ROLE OF THE IMMUNE RESPONSE IN BREAST CANCER

Breast cancer progression proceeds through well-defined stages starting from hyperplasia to ductal carcinoma *in situ *(DCIS), transition to invasive ductal carcinoma (IDC) and development of metastatic disease. Dynamic interactions between cancer and immune cells influence each of these processes (Joyce and Pollard, 2009). Innate and adaptive immune cells polarize into numerous lineages with contradicting roles during cancer progression. The neoplastic process favors subtypes that exert a chronic inflammatory and immunosuppressive state (de Visser et al., 2006).

### CYTOTOXIC T LYMPHOCYTES

Cytotoxic T lymphocytes (CTLs) are CD8^+^ T cells that mediate anti-tumor immunity. In concert with CD8, antigen-specific T cell receptors bind to peptide-loaded major histocompatibility complex (MHC) class I molecules on the surface of tumor cells. This activates CTLs to kill tumor cells by perforin-mediated permeabilization of the plasma membrane and delivery of granzyme-containing granules into the cytoplasm. Granzymes are serine proteases that promote cell death through multiple mechanisms. Granzyme A increases oxidative stress by decreasing the mitochondrial membrane potential. In contrast, Granzyme B activates the apoptotic machinery by cleaving numerous cellular targets. These include: (1) direct cleavage and activation of effector caspases (Caspase-3; Caspase-7) and (2) cleavage of Bid, a pro-apoptotic Bcl2 family member, which translocates to and oligomerizes in the outer mitochondrial membrane, which stimulates cytochrome c release to promote apoptosome-mediated Caspase-9 activation. Emerging evidence also suggests a novel pro-inflammatory role for extracellular granzymes released from target cells (Cullen et al., 2010). Along with natural killer (NK) cells, CTLs are primarily responsible for immune surveillance (Schirrmacher, 2001). Retrospective studies reveal a strong relationship between high CTL recruitment and good outcome in breast cancer patients (Finak et al., 2008; DeNardo et al., 2009).

### MACROPHAGES/MYELOID-DERIVED SUPPRESSOR CELLS

While M1-polarized macrophages possess anti-tumorigenic properties, M2-type tumor-associated macrophages (TAMs) facilitate tumorigenesis through their ability to secrete cytokines, growth factors, and proteases that induce immunosuppression, angiogenesis, and re-modeling of the extracellular matrix (Allavena et al., 2008). Increased numbers of intra-tumoral macrophages is indicative of poor outcome in breast cancer patients (Leek et al., 2000; Tsutsui et al., 2005). Myeloid-derived suppressor cells (MDSCs) are a heterogeneous population of progenitor cells within the myeloid lineage. While these cells normally reside in the bone marrow and differentiate prior to entering the bloodstream, cancer cells induce the production of cytokines that stimulate myelopoiesis and block their further differentiation (Gabrilovich and Nagaraj, 2009). Elevated circulating MDSC levels correlate with increasing grade and malignancy in breast cancer patients (Diaz-Montero et al., 2009). These cells exhibit potent immunosuppressive activity (Gabrilovich and Nagaraj, 2009).

### HELPER T CELLS

CD4^+^ helper T (Th) cells differentiate into numerous sub lineages that perform distinct biological functions (Zhu and Paul, 2008). *Th1* cells elicit an anti-tumor immune response by secreting high levels of interferon (IFN)γ and interleukin (IL)-2, which induce M1 macrophage polarization and promote CTL proliferation/survival, respectively (de Visser et al., 2006). Regulatory T cells (*Tregs*) establish immune suppression by inhibiting CTL/NK cell function via diverse mechanisms. These include: (1) competitive binding of IL-2 to the high affinity IL-2 receptor (CD25); this deprives effector T cells of IL-2, leading to apoptosis; (2) secretion of immunosuppressive cytokines [IL-10 and transforming growth factor beta (TGFβ)], which impairs antigen-activated CTL clonal expansion; and (3) upregulation of cell surface inhibitory molecules cytotoxic T lymphocyte associated protein 4 (CTLA-4) that inhibit dendritic cell maturation (Vignali et al., 2008). *Th2* cells induce B cell activation and differentiation, which stimulates the production of inflammatory cytokines. Th2 cells also impair CTL activation by (1) inhibiting Th1 differentiation, and (2) secreting immunosuppressive cytokines such as IL-10 and TGFβ (de Visser et al., 2006; Johansson et al., 2008). B and T cell infiltrates progressively increase during breast cancer progression, and a high CD4/CD8 T cell ratio is associated with increased lymph node positivity and poor outcome (DeNardo and Coussens, 2007). This is recapitulated in murine breast cancer models where the Th2 response induces M2-macrophage polarization and metastasis (DeNardo et al., 2009). *Th17 *cells, which induce inflammation, represent a new paradigm in cancer biology. While clinical studies suggest that Th17 cell infiltration is associated with Th1-driven anti-tumor immunity in breast cancer (Yang et al., 2012), others demonstrate that Th17-mediated chronic inflammation potentiates breast cancer progression in transgenic mouse models of the disease (Novitskiy et al., 2011).

## MECHANISMS TO ESTABLISH BREAST CANCER IMMUNE SUPPRESSION

During the early stages of the neoplastic process, immune cells recognize and destroy tumors through a process termed immune surveillance (Ostrand-Rosenberg, 2008). Escaping tumor cells proceed to an equilibrium phase whereby continuous interactions between cancer and immune cells prevent tumor growth (Schirrmacher, 2001). Both stages are controlled via the action of CTL and NK cells. In the final phase, cancer cells acquire the ability to escape these immune-protective effects through a process termed immune suppression and form clinically detectable tumors (Dunn et al., 2002). Tumor cells employ multiple mechanisms to impair CTL infiltration, activation or survival in order to establish immunosuppression:

### CHEMOKINE/CYTOKINE PRODUCTION

Cancer cells promote selective chemotaxis of polarized immune cells that confer pro-tumorigenic properties. This is achieved via the secretion of chemokines, which establish a chemical gradient for immune cell types expressing their cognate receptor. Numerous chemokines have been implicated in breast cancer progression (Roussos et al., 2011). Breast cancer cells secrete CCL17 and CCL22, which recruit CCR4-expressing Treg and Th2 cells (Faget et al., 2011). Elevated CCL22 expression is associated with increased Treg recruitment into primary DCIS and IDC lesions, suggesting that Tregs may contribute to early breast cancer immunosuppression (Ohara et al., 2009). Breast cancer cells are also rich sources of immunosuppressive cytokines, including IL-10 and TGFβ, which directly promote CTL anergy (Koli and Arteaga, 1996; Hamidullah et al., 2012). IL-10 is upregulated in ER/PR-negative and HER2-positive breast tumors (Ohara et al., 2009).

In contrast, trafficking of antigen-activated CXCR3-expressing NK, CTL, and/or Th1 cells into tumor tissue is promoted via the action of CXCL9 and CXCL10, two IFNγ-inducible chemokines (Farber, 1997; Thapa et al., 2008). CXCL9 overexpression in breast cancer cells increases T cell infiltration, decreases tumor growth and prolongs survival of immunocompetent but not immunocompromised mice (Walser et al., 2007). Moreover, CXCL9 overexpression in primary breast cancers is associated with increased T cell infiltration and enhanced chemotherapy responsiveness (Specht et al., 2009; Denkert et al., 2010). This suggests that CXCL9 promotes Th1/CTL-mediated anti-tumor immunity.

### ANTIGEN PROCESSING AND PRESENTATION

Major histocompatibility complex class I proteins, along with components of the antigen processing and presentation (APP) machinery are ubiquitously expressed in adult tissues. MHC class I-restricted Ag presentation on the surface of breast cancer cells contributes to immune surveillance through the activation of CTLs, leading to tumor cell lysis. APP is a complex process. Ubiquitinated proteins are cleaved by a multi-subunit complex called the proteasome. Peptides are transported into the endoplasmic reticulum via the transporter associated with antigen processing (TAP1, TAP2) heterodimer where they are processed by ER aminopeptidase-associated with antigen processing (ERAP1, ERAP2) proteins. Also in the ER, the MHC class I heavy human leukocyte antigen (HLA) and light chains (β2-microglobulin) assemble into a functional MHC class I complex. Finally, the peptides are loaded onto MHC class I complexes and translocate to the cell surface for presentation to CTLs (Seliger, 2008). Several proteins involved in APP are deleted, mutated or downregulated in breast cancer cells (Seliger, 2008). ErbB2 signaling reduces expression levels of numerous components of the APP machinery (Herrmann et al., 2004). Moreover, ErbB2-driven mammary tumors display reduced surface MHC class I levels in transgenic mice (Lollini et al., 1998). Finally, up to 50% of primary breast cancers have reduced surface MHC class I expression, correlating with increasing grade (Vitale et al., 1998; Gobbi et al., 2004).

### DIMINISHED CYTOTOXIC T LYMPOCYTE EFFECTOR FUNCTION

The CD28 family of co-receptors are expressed on the surface of T cells and transduce co-stimulatory or inhibitory signals following antigen activation. Programmed cell death 1 (PD1) is an inhibitory member that is expressed on the surface of antigen-activated CTLs. Upon binding to its cognate ligand programmed cell death ligand 1 (PD-L1) on the surface of antigen presenting cells, PD1 transduces a signal that reduces CTL proliferation and cytolytic activity, eventually leading to CTL apoptosis (Flies et al., 2011). PD-L1 is elevated on the surface of breast cancer cells, correlating with a high mitotic index, increasing grade, and ER/PR negativity (Ghebeh et al., 2006, 2007).

## ANTI-TUMOR IMMUNITY IN POOR OUTCOME BREAST CANCERS

An increasing body of evidence is emerging that Th1/CTL-driven anti-tumor immunity plays a critical role in contributing to enhanced patient outcome and therapeutic responsiveness in breast cancer patients, particularly within the poor outcome HER2 and basal subtypes.

### PATIENT OUTCOME

Gene expression profiling has revolutionized how we study breast cancer. The Park laboratory described the first stromal-derived prognostic predictor (SDPP) for breast cancer patients (Finak et al., 2008). Subsets of genes within the SDPP include a Th1/CTL signature that predicts reduced mortality in breast cancer patients, independently of clinical variables such as grade, lymph node positivity, ER, and HER2 status (Finak et al., 2008). We independently identified an immune signature, comprising B and T cell-specific genes, which is elevated in mouse mammary tumors that are impaired in ErbB2 signaling relative to wild-type tumors. This signature functions as an independent prognostic marker of good outcome in HER2 and basal breast cancer patients but not in the luminal subtypes (Ursini-Siegel et al., 2010). Moreover, a Th2 signature is selectively enriched in poor outcome basal breast cancers and an elevated Th1/Th2 ratio is highly predictive of good outcome in HER2 and basal breast cancer patients (Kristensen et al., 2012). These observations are consistent with studies demonstrating that high intra-tumoral CTL infiltration is enriched in ER^-^/PR^-^, and/or basal breast tumors and strongly correlates with favorable prognosis in this subset (Baker et al., 2011; Mahmoud et al., 2011; Liu et al., 2012). Paradoxically, increased CTL infiltration is associated with inferior outcome in low grade ER^+^ breast cancer patients (Baker et al., 2011). Taken together, these observations provide strong clinical rationale to study how poor outcome breast cancers impair Th1/CTL-driven immunity within the HER2 and basal subtypes.

### THERAPEUTIC RESPONSIVENESS

Trastuzumab is a monoclonal antibody specific for ErbB2, which when coupled with chemotherapy significantly improves outcome of early stage HER2^+^ breast cancer patients. The therapeutic efficacy of trastuzumab relies both on its ability to elicit anti-tumor immunity by engaging FcR-mediated cytotoxicity and by blocking ErbB2-driven signal transduction (Hudis, 2007). Using pre-clinical mouse models, it has become clear that the therapeutic effect of anti-ErbB2 monoclonal antibodies relies on innate and adaptive immunity, including NK/CTL activity and IFNγ responsiveness (Park et al., 2010; Stagg et al., 2011). Indeed, trastuzumab sensitizes HER2-overexpressing tumors to CTL-mediated lysis (Kono et al., 2004). Moreover, treatment of ErbB2-driven mouse mammary tumors with ErbB2 neutralizing antibodies together with v-akt murine thymoma viral oncogene homolog 1 (AKT) inhibitors results in increased CTL infiltration and delayed tumor outgrowth (Wang et al., 2012). Finally, HER2^+^ breast tumors treated with trastuzumab plus anthracyclines exhibit increased Th1/CTL-driven immunity relative to chemotherapy alone (Ladoire et al., 2011). This suggests that trastuzumab elicits anti-tumor immunity by attenuating breast cancer immune suppression.

Emerging evidence suggests that chemotherapies function, in part, by altering the immune microenvironment. It has been demonstrated that subsets of HER2 and basal breast cancers are highly sensitive to neo-adjuvant chemotherapy, displaying a four- to sixfold increase in pathologic complete response (CR) relative to luminal tumors. Despite this fact, a large percentage of patients within the HER2 and basal subtypes experience relapse (Carey et al., 2007). Recent studies have shown that in ER negative breast cancer, including the HER2 and basal subtypes, the presence of tumor-infiltrating CTLs correlates with anthracycline responsiveness and prolonged patient survival compared to patients without adjuvant chemotherapy (West et al., 2011). Finally, a recent study further identified an immune signature (CD68^high^; CD4^high^; CD8^low^), which is indicative of high macrophage density and low CTL infiltration that predicts poor recurrence free survival in node-positive breast cancer patients. Moreover, macrophage infiltration is significantly increased in breast tumors from patients who underwent adjuvant chemotherapy compared to women without any pre-operative intervention (DeNardo et al., 2011). Macrophage infiltration is also enriched following paclitaxel treatment and contributes to reduced chemosensitivity in the mouse mammary tumor virus/polyoma virus middle T (MMTV/PyvMT) breast cancer mouse model. Finally, depletion of tumor associated macrophages increases CTL infiltration and enhances anti-tumor immunity in MMTV/PyvMT mice in response to paclitaxel (DeNardo et al., 2011). Take together; this suggests that chemotherapies stimulate anti-tumor immunity.

## RECEPTOR TYROSINE KINASES INHIBIT ANTI-TUMOR IMMUNITY

In addition to these clinical correlations, functional studies in murine breast cancer models have demonstrated an important role for RTK signaling in blocking anti-tumor immunity and promoting disease progression. The laboratory of Dr. Brad Nelson has engineered the first immunological mouse model for ErbB2-driven breast cancer. MMTV/ErbB2 transgenic mice were generated using a modified version of ErbB2 that was tagged with T cell epitopes from the model antigen ovalbumin (OVA). A large panel of tumor cell lines [referred to as neu ovalbumin peptide (NOP) lines] from spontaneous ErbB2/OVA-expressing mammary tumors were established. When implanted in host mice and challenged by adoptive transfer of OVA-specific CTLs, NOP tumors show highly reproducible responses. Some tumor lines demonstrate a CR, whereas others demonstrate a partial response (PR) or progressive disease (PD; Wall et al., 2007; Martin et al., 2010). This panel of cell lines provides a unique and powerful system to define and manipulate the factors that dictate the immunological sensitivity of mammary tumors *in vivo*.

More recently, we established a unique transgenic mouse model of ErbB2-driven breast cancer in which mammary epithelial expression of ErbB2/Neu is coupled to the Cre recombinase using an intervening internal ribosome entry site (IRES) element neu-ires-cre (NIC; Ursini-Siegel et al., 2008). MMTV/NIC mice were employed to study the importance of the ShcA adaptor during mammary tumorigenesis. ShcA transduces Grb2-dependent and -independent signals downstream of RTKs to regulate proliferation, survival, angiogenesis, invasion, and metastasis (Northey et al., 2008; Ursini-Siegel and Muller, 2008; Ursini-Siegel et al., 2008). The majority of RTKs that are expressed in breast cancer cells, including ErbB2 and Met, couple to ShcA. Increased ShcA signaling predicts nodal status and relapse in breast cancer patients (Davol et al., 2003; Frackelton et al., 2006). Moreover, high ShcA levels are enriched within the HER2 (ErbB2^+^) and basal (ER^-^/PR^-^/ErbB2^-^) subtypes and associate with poor outcome in breast cancer patients (Ursini-Siegel et al., 2010). Using breast cancer mouse models that ablate ShcA expression (NIC/ShcA^fl/fl^), we defined a causal role for ShcA in establishing immune suppression. ShcA loss in the mammary epithelium severely delays mammary tumor onset (Ursini-Siegel et al., 2010). ShcA-deficient pre-neoplastic lesions display enhanced CTL-driven, anti-tumor immune responses, relative to their wild-type counterparts. Emerging ShcA-deficient tumors bypass these anti-tumorigenic conditions by polarizing the immune response to favor a Th2-driven immunity, which establishes an immunosuppressive state (Ursini-Siegel et al., 2010). Thus, RTKs, such as ErbB2, engage ShcA to establish immunosuppression and facilitate the emergence of clinically detectable tumors.

## CAN RTK INHIBITORS SENSITIZE TUMORS TO BREAST CANCER IMMUNOTHERAPY?

The immune response is an attractive target for therapeutic intervention in treating breast cancer. Several immune-based therapies have been tested in clinical trials. These include: (1) DNA, peptide and protein vaccines to tumor antigens such as HER2 and MUC1, (2) autologous dendritic cell-based vaccines, (3) cytokine therapies, including granulocyte macrophage-colony stimulating factor (GM-CSF), as immune adjuvants, and (4) compounds that block Treg function. Unfortunately, such trials have met with limited success in treating breast cancer patients. It is likely that local immunosuppression contributes to this poor success rate (Wright, 2012). One approach to improving the efficacy of breast cancer immunotherapy is by manipulating innate and adaptive immune cells to increase CTL infiltration and/or cytotoxicity (DeNardo et al., 2011; Manjili and Payne, 2012). Published observations also suggest a critical role for RTKs in establishing breast cancer immune suppression (Ursini-Siegel et al., 2010). An increased Th1/CTL response is associated with chemosensitivity and increased survival in HER2 and basal breast cancer patients (Finak et al., 2008; DeNardo et al., 2009; Ursini-Siegel et al., 2010; West et al., 2011). Finally, several RTK inhibitors are approved (trastuzumab) or in clinical trials (Lapatinib, Erlotinib, Tivantinib) to treat breast cancer patients within these poor outcome subtypes. This raises the intriguing possibility that pharmacological RTK inhibitors, alone or in combination with chemotherapy, provide a therapeutic window to sensitize these poor outcome tumors to immune-based therapies.

## Conflict of Interest Statement

The author declares that the research was conducted in the absence of any commercial or financial relationships that could be construed as a potential conflict of interest.
